# Reversible photo-induced trap formation in mixed-halide hybrid perovskites for photovoltaics†
†Electronic supplementary information (ESI) available: Experimental details, PL, PDS spectra and XRD patterns. See DOI: 10.1039/c4sc03141e



**DOI:** 10.1039/c4sc03141e

**Published:** 2014-11-04

**Authors:** Eric T. Hoke, Daniel J. Slotcavage, Emma R. Dohner, Andrea R. Bowring, Hemamala I. Karunadasa, Michael D. McGehee

**Affiliations:** a Department of Materials Science and Engineering , Stanford University , 476 Lomita Mall , Stanford , California 94305 , USA . Email: mmcgehee@stanford.edu; b Department of Chemistry , Stanford University , 337 Campus Drive , Stanford , California 94305 , USA . Email: hemamala@stanford.edu

## Abstract

We report on reversible, light-induced transformations in (CH_3_NH_3_)Pb(Br_*x*_I_1–*x*_)_3_. Photoluminescence (PL) spectra of these perovskites develop a new, red-shifted peak at 1.68 eV that grows in intensity under constant, 1-sun illumination in less than a minute. This is accompanied by an increase in sub-bandgap absorption at ∼1.7 eV, indicating the formation of luminescent trap states. Light soaking causes a splitting of X-ray diffraction (XRD) peaks, suggesting segregation into two crystalline phases. Surprisingly, these photo-induced changes are fully reversible; the XRD patterns and the PL and absorption spectra revert to their initial states after the materials are left for a few minutes in the dark. We speculate that photoexcitation may cause halide segregation into iodide-rich minority and bromide-enriched majority domains, the former acting as a recombination center trap. This instability may limit achievable voltages from some mixed-halide perovskite solar cells and could have implications for the photostability of halide perovskites used in optoelectronics.

## Abstract 2

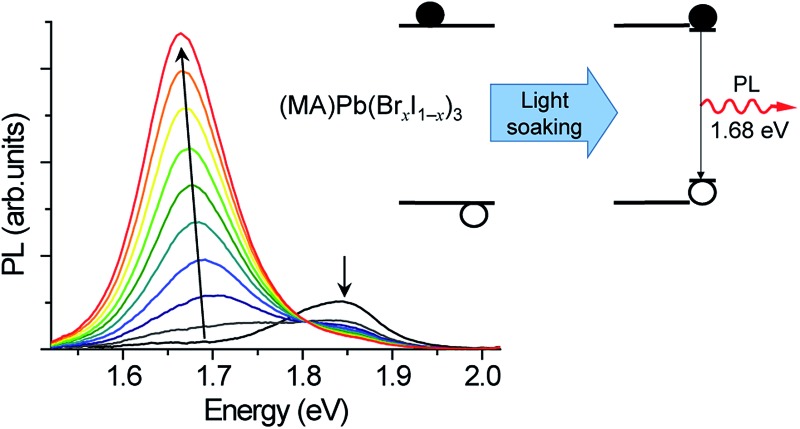
A reversible photo-induced instability has been found in mixed-halide photovoltaic perovskites that limits the open circuit voltage in solar cells.

## Introduction

Hybrid perovskites have attracted significant attention over the past few years as absorbers for solar cells^1–4^ with power conversion efficiencies (PCEs) exceeding 15%.^5–8^ One attractive attribute of hybrid perovskites as photovoltaic absorbers is the ability to continuously tune the absorption onset by alloying different halides into the structure. For example, the bandgap of (MA)Pb(Br_*x*_I_1–*x*_)_3_ (MA = CH_3_NH_3_) can be continuously tuned over the range 1.6–2.3 eV,^9^ making these materials suitable both for single-junction solar cells and for the larger bandgap absorber of tandem solar cells. Photovoltaic devices containing (MA)Pb(Br_*x*_I_1–*x*_)_3_ have demonstrated PCEs of 4–16% for a wide range of halide ratios,^8–12^ and an open circuit voltage (*V*_OC_) of 1.5 V has been achieved using the largest bandgap perovskite of this family: (MA)PbBr_3_.^13^ Although solar cells containing (MA)PbI_3_ have obtained *V*_OC_'s of up to 1.15 V,^14^ solar cells with mixed-halide perovskites have so far not produced the larger *V*_OC_'s that may be expected from their larger bandgaps. Several groups have reported a decrease in *V*_OC_, despite the increase in optical band gap, in (R)Pb(Br_*x*_I_1–*x*_)_3_ (R = CH_3_NH_3_ or HC(NH_2_)_2_) absorbers for *x* > 0.25.^9–11,15^

We examined the optical properties of (MA)Pb(Br_*x*_I_1–*x*_)_3_ thin films to understand the poor voltage performance of solar cells with the bromide-rich alloys. We find that the photoluminescence (PL) spectra of these materials discretely red-shift to ∼1.68 eV under illumination intensities of less than 1 sun in less than a minute at room temperature. This red-shift is accompanied by an increase in absorption between 1.68 eV and the bandgap. X-ray diffraction (XRD) patterns of the thin films show that the original peaks split upon illumination and revert back to their original line shape after a few minutes in the dark. Our observations so far are consistent with light-induced segregation of the mixed-halide alloy. We hypothesize that photoexcitation induces halide migration, which results in lower-bandgap, iodide-rich domains that pin the PL and *V*_OC_ at a lower energy compared to the alloy.

## Results and discussion

**Fig. 1:**
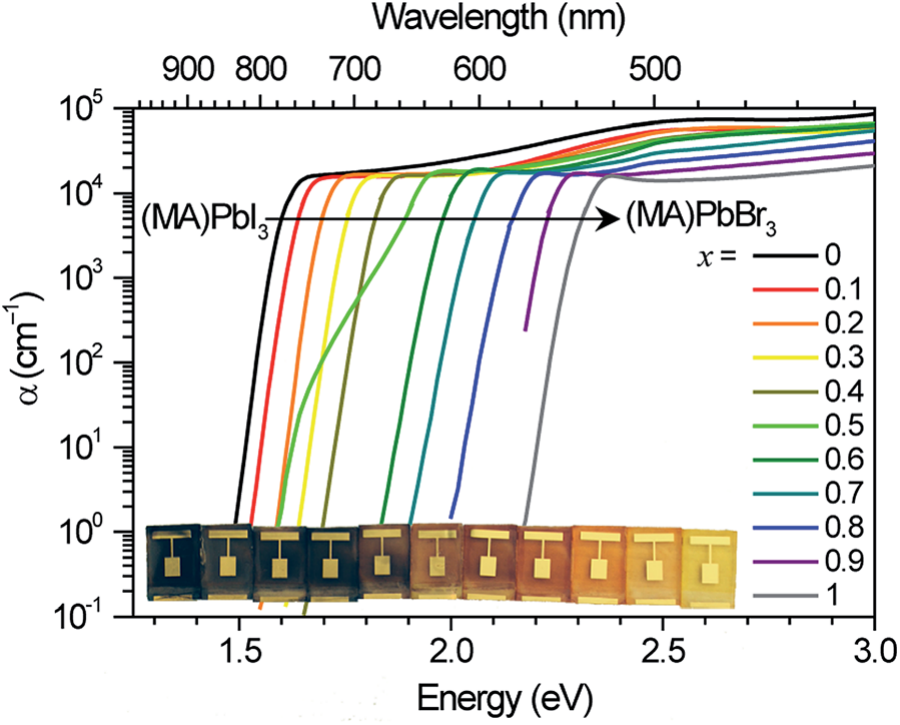
Absorption coefficient of (MA)Pb(Br_*x*_I_1–*x*_)_3_ measured by diffuse spectral reflection and transmission measurements on thin films and photocurrent spectroscopy of solar cells. Inset: photograph of (MA)Pb(Br_*x*_I_1–*x*_)_3_ photovoltaic devices from *x* = 0 to *x* = 1 (left to right).

We measured the absorption coefficients of (MA)Pb(Br_*x*_I_1–*x*_)_3_ over the full range of compositions to characterize band-edge states and optical bandgaps (Fig. 1). Thin films of (MA)Pb(Br_*x*_I_1–*x*_)_3_ were spun from equimolar mixtures of 0.55 M PbI_2_ + (MA)I and PbBr_2_ + (MA)Br solutions in dimethyl formamide and annealed for 5 minutes at 100 °C in dry air. The phase purity of the films was confirmed with XRD; their pseudo-cubic lattice parameters agree with previous reports (ESI, Fig. S1†).^9^ Photocurrent spectroscopy (*i.e.*, external quantum efficiency) measurements were performed on (MA)Pb(Br_*x*_I_1–*x*_)_3_ photovoltaic devices using a lock-in amplifier to measure weak absorption from band-edge states. At these weakly absorbed wavelengths, the photocurrent is proportional to the perovskite layer absorption coefficient. We combined these measurements with diffuse transmission and reflection measurements on films of varying thickness to obtain the full absorption spectra. These spectra continuously blue-shift upon increasing bromide content as previously reported.^9^ All perovskites in this family (except for *x* = 0.5) have strong absorption onsets, yielding absorption coefficients above 1 × 10^4^ cm^–1^ at energies only 0.1 eV above the bandgap. This property is highly desirable for thin-film photovoltaic absorbers. These absorption onsets correspond to Urbach energies in the range 12–17 meV. These values are similar to the reported value of 15 meV for (MA)PbI_3_,^16,17^ indicating that mixed halide films are homogeneous in composition. In contrast, the *x* = 0.5 thin films exhibit a more gradual absorption onset, suggesting the presence of minority, iodide-rich domains (*x* ∼ 0.2). Photothermal deflection spectroscopy (PDS) measurements corroborate the sharp absorption onset for all compositions except for *x* = 0.5 (Fig. S2†).

**Fig. 2:**
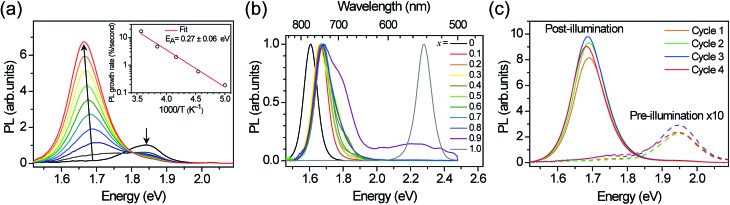
(a) Photoluminescence (PL) spectra of an *x* = 0.4 thin film over 45 s in 5 s increments under 457 nm, 15 mW cm^–2^ light at 300 K. Inset: temperature dependence of initial PL growth rate. (b) Normalized PL spectra of (MA)Pb(Br_*x*_I_1–*x*_)_3_ thin films after illuminating for 5–10 minutes with 10–100 mW cm^–2^, 457 nm light. (c) PL spectra of an *x* = 0.6 thin film after sequential cycles of illumination for 2 minutes (457 nm, 15 mW cm^–2^) followed by 5 minutes in the dark.

The initial PL spectra for (MA)Pb(Br_*x*_I_1–*x*_)_3_ at low illumination intensities also continuously blue-shift upon increasing bromide content (Fig. S3†). However, for perovskites with 0.2 < *x* < 1 we find that an additional PL peak forms at ∼1.68 eV and grows in intensity under continuous illumination (Fig. 2a). The position of this new peak is independent of halide composition and bandgap (Fig. 2b). After less than a minute of continuous visible-light soaking (argon ion laser, 457 nm, 15 mW cm^–2^) the PL intensity from the new low-energy peak becomes more than an order of magnitude more intense than the original peak (Fig. 2c). Films with higher iodide content exhibited higher initial luminescence efficiencies and required more light soaking for the new PL feature to dominate the original PL. We find that this PL spectral change is not dependent upon the spectrum or coherence of the light source and that it occurs as long as the light is absorbed by the perovskite: we observed similar changes in the PL spectra upon light soaking with various white LEDs, 375 nm and 457 nm laser excitation, and red LED excitation (∼637 nm) for the perovskites that absorb this wavelength. Notably, these changes are reversible; the original PL spectra return after the materials are left in the dark for 5 minutes. Moreover, the spectra can be repeatedly cycled between these two states by turning on and off the excitation light (Fig. 2c and S4†).

**Fig. 3:**
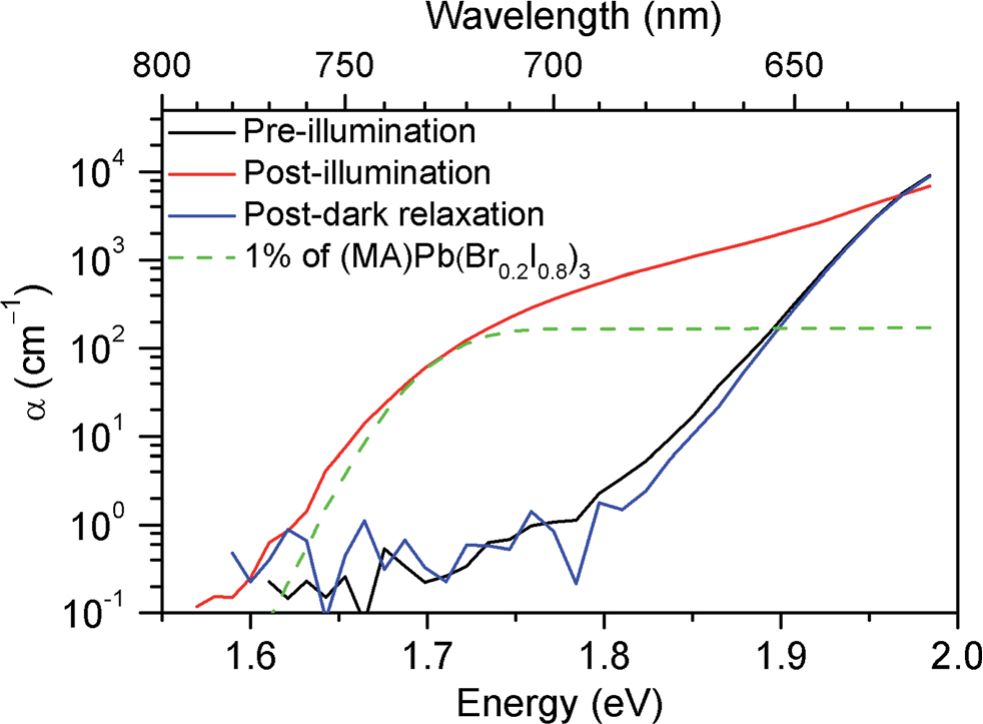
Absorption spectra of an *x* = 0.6 film before (black) and after (red) white-light soaking for 5 minutes at 100 mW cm^–2^, and after 1 h in the dark (blue). A scaled absorption spectrum of an *x* = 0.2 film (dashed green) is shown for comparison.

To understand the origin of the new PL feature, we performed photocurrent spectroscopy measurements on photovoltaic devices containing (MA)Pb(Br_*x*_I_1–*x*_)_3_ before and after light soaking to characterize absorption from band-edge states (Fig. 3). A new absorption shoulder forms around 1.7 eV after light soaking, which completely disappears after the devices are left in the dark for 1 h. We speculate that these new PL and absorption features in the light-soaked mixed-halide perovskites are due to the formation of small, iodide-enriched domains with a lower bandgap compared to the alloy. The absorption shoulder in the light-soaked *x* = 0.6 alloy has an absorption coefficient similar to the expected value if ∼1% of the material converted into the *x* = 0.2 perovskite (Fig. 3). The observations of this absorption shoulder and additional PL peak at 1.68 eV were recently reported for (MA)Pb(Br_0.4_I_0.6_)_3_ and were attributed to the existence of multiple phases.^17^ However, the role of light soaking in producing these features was not examined.

Minority domains with the highest iodide content can dominate the PL spectra even at low volume fractions because photogenerated carriers relax into lower energy states and predominately emit from the lowest bandgap domains (the iodide-rich domains, in this case). The large increase in overall PL intensity during light soaking suggests that these defect domains have a higher luminescence efficiency than the rest of the perovskite film. This may be a consequence of the domains acting as carrier traps, concentrating and facilitating radiative electron–hole recombination, similar to how quantum wells and emissive impurities can increase the quantum efficiencies of III–V semiconductor LEDs and organic LEDs, respectively. The PL quantum efficiency of (MA)PbI_3_ perovskite thin films has been reported to be quite high—in excess of 20%.^18,19^

**Fig. 4:**
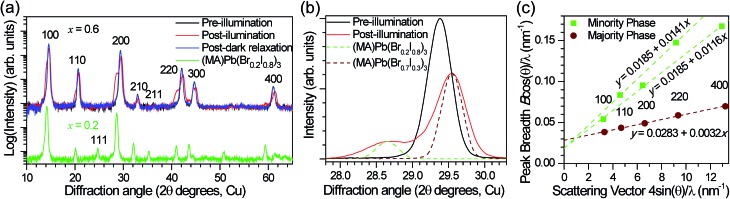
(a) XRD pattern of an *x* = 0.6 film before (black) and after (red) white-light soaking for 5 minutes at ∼50 mW cm^–2^, and after 2 h in the dark (blue). The XRD pattern of an *x* = 0.2 film (green) is offset for comparison. (b) The 200 XRD peak of an *x* = 0.6 film before (black) and after (red) white-light soaking for 5 minutes at ∼50 mW cm^–2^. XRD patterns of an *x* = 0.2 film (dashed green) and an *x* = 0.7 film (dashed brown) are included for comparison. (c) Williamson–Hall plot of the XRD peak full width at half maximum (*B*) for the minority (green, larger lattice spacing) and majority (brown, smaller lattice spacing) phases observed in (MA)Pb(Br_0.6_I_0.4_)_3_ (*x* = 0.6) thin films under illumination. *θ* is the diffraction angle and *λ* = 1.54060 Å (copper Kα_1_) is the X-ray wavelength. The points are labeled with their crystallographic indices. Linear regressions to the data are plotted and the equations are listed. The minority phase was fit assuming the same crystallite size in the 100 and 110 directions but different amounts of strain disorder.

In order to reversibly create iodide-rich domains, the bromide concentration should be slightly enhanced elsewhere in the films. To test this hypothesis, we performed XRD measurements on *x* = 0.6 thin films before and after light soaking. We observe splitting of all XRD diffraction peaks with light soaking, and regeneration of the original sharp diffraction patterns after the films are left in the dark (Fig. 4a). Since perovskites with higher bromide content have a smaller lattice constant than those with higher iodide content (Fig. S1†), this splitting is consistent with the presence of a minority phase with significantly enhanced (*x* ∼ 0.2) iodide content and a majority phase with slightly enhanced bromide content (*x* ∼ 0.7) compared to the original material (Fig. 4b). These phase compositions would suggest that the minority phase is about 20% of the material in these particular samples. If we compare the magnitude of XRD intensity from the two phases, we estimate that the minority phase makes up 23% of the material, after accounting for differences in structure factor for the two hypothesized phases. These values are substantially higher than the 1% minority phase estimated from the absorption measurements by photocurrent spectroscopy on mesoporous devices (Fig. 3). We suggest that differences in morphology between the mesoporous devices and planar thin films may be responsible for the different minority phase yields under similar illumination conditions. Assuming that the creation of the minority phase is proportional to the light dosage, the XRD measurements suggest that for this particular sample at room temperature, on average roughly 200 absorbed photons cause one cubic unit cell (containing one Pb atom) of the minority iodide-rich phase to form. We note that other samples with different halide compositions (Fig. S5†), or processed differently did not form as much of the minority phase under the same illumination conditions.

Assuming that the XRD peak full width at half maximum (*B*) is primarily governed by crystallite size and strain, the *y*-intercept of a linear fit of the peak breadth *versus* scattering vector on a Williamson–Hall plot (Fig. 4c) is inversely proportional to the crystallite grain diameter and the slope is equal to the average uncorrelated strain.^20^ We estimate a crystallite size of 33 ± 1 nm for the majority phase and 51 ± 5 nm for the minority phase from the Scherrer equation (see ESI† materials for calculation details). This provides a lower bound for the size of the phase segregated domains, which may contain several crystallites. Additional peak broadening from compositional inhomogeneity may also produce an underestimation in this calculation. The minority domains exhibit anisotropic strain disorder (1.16 ± 0.02% in the 100 direction and 1.41 ± 0.03% in the 110 direction) and have significantly more strain disorder than the majority domains (0.30 ± 0.01%, Fig. 4c). Other mixed-halide compositions also exhibit an asymmetric splitting of the 200 reflection after light soaking (Fig. S5†). All compositions (0.2 < *x* < 1) show increased scattering intensity at ∼28.5°, further suggesting the presence of iodide-enriched (*x* = 0.2) domains irrespective of the initial stoichiometry.

We considered the possibility that photo-induced lattice expansion could also produce the observed reversible structural changes and photochromic responses. *Ab initio* calculations have recently suggested that photoexcitation may reduce hydrogen bonding in (MA)PbI_3_, resulting in a slight unit-cell expansion^21^ and bandgap reduction.^22^ However, this is inconsistent with the observed XRD peak splitting, which indicates domains with both smaller and larger lattice constants. We also don't see significant spectral or structural changes upon illumination of (MA)PbI_3_ or (MA)PbBr_3_ thin films (Fig. S3, 2b, and S5†), where halide segregation cannot occur.

**Fig. 5:**
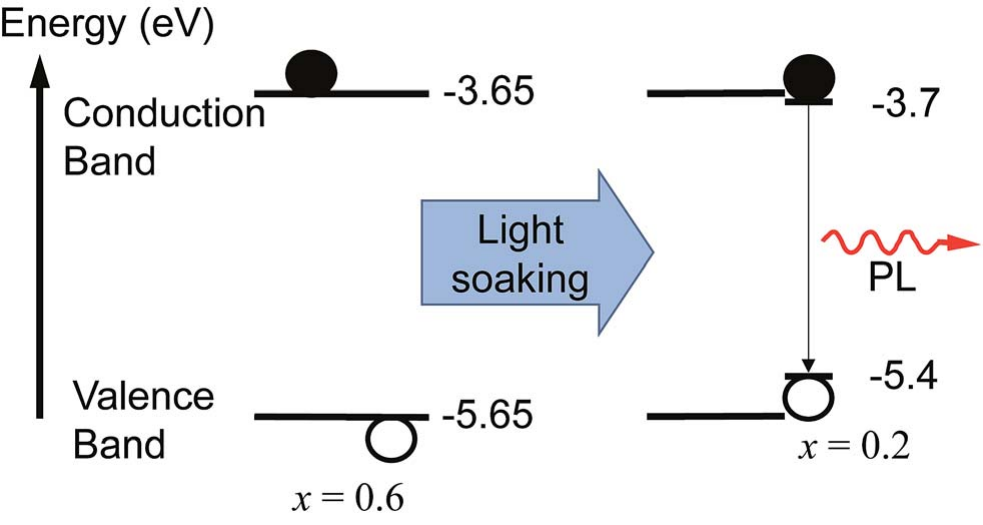
Schematic of the proposed mechanism for photo-induced trap formation through halide segregation. Photogenerated holes or excitons may stabilize the formation of iodide-enriched domains which then dominate the photoluminescence. The valence band (VB) and conduction band (CB) energies with respect to vacuum were estimated by interpolation of published values obtained from ultraviolet photoemission spectroscopy (UPS) and inverse photoemission spectroscopy (IPES) for the endpoint stoichiometries.^35^

We monitored the PL spectral evolution under constant illumination at different temperatures to study the kinetics of this conversion. The low-energy PL peaks also form below room temperature (200–280 K), indicating that the spectral change is due to photoexcitation and not heating from the light. The initial growth rate in the low-energy PL peak follows Arrhenius behavior with an activation energy of 0.27 ± 0.06 eV (Fig. 2a inset). This value is similar to the activation energies attributed to halide migration in the perovskites CsPbCl_3_, CsPbBr_3_, KMnCl_3_, CuCdCl_3_, KPbI_3_, CuSnI_3_, and CuPbI_3_, which span the range 0.25–0.39 eV.^23–26^ Light-induced halide migration has also been reported to occur in metal halides such as PbBr_2_ and PbI_2_,^27,28^ and is the basis for latent image formation in photography using AgI.^29^ While halide mobilities in hybrid lead-halide perovskites have not yet been reported, ion conductivities of 7 × 10^–8^ and 3 × 10^–9^ S cm^–1^ have been reported for KPbI_3_ and CuPbI_3_, respectively.^24^ Since the (MA)Pb(Br_*x*_I_1–*x*_)_3_ valence band is dominated by contribution from the halide p orbitals,^30^ we speculate that formation of iodide-enriched domains stabilizes holes, which could provide a driving enthalpy for halide segregation under illumination (Fig. 5). When these trapped holes are filled, entropy and lattice strain may cause the phase segregated material to relax back to the well-mixed alloy. Light-induced, reversible structural changes in PbBr_2_ have been attributed to self-trapping of such photogenerated holes.^31^ Alternatively, since iodide-rich domains have a smaller bandgap, they lower the energy of excitons, which could drive halide segregation. It is not yet clear whether these structural and spectroscopic changes can be induced by electrical excitation. Spectrally stable red-emitting (MA)PbBr_2_I LEDs have been recently demonstrated.^32^ This suggests that the application of an electrical bias might not produce the large changes in emission that are observed under photoexcitation and that photogenerated excited states may play an important role in the transformation mechanism. It is also not yet clear why photo-induced defects resemble the *x* = 0.2 perovskite for a range of bulk perovskite stoichiometries.

This ion-transport mechanism suggests that crystallite size and quality should influence its kinetics. Accordingly, we have seen variations between samples in the rate of their light-induced changes. We see the growth of the low-energy PL even in single crystals of (MA)Pb(Br_*x*_I_1–*x*_)_3_ (Fig. S6†), indicating that significant grain boundaries or surface defects are not required for this transformation. We have also observed this new phase upon light exposure (for both white LED and 457 nm excitation at 10–100 mW cm^–2^) in (MA)Pb(Br_*x*_I_1–*x*_)_3_ thin films formed from a PbCl_2_ precursor,^10^ sequentially-deposited dip-converted^11^ and vapor-converted^33^ thin films, and in (HC(NH_2_)_2_)Pb(Br_*x*_I_1–*x*_)_3_ thin films (Fig. S7†),^15^ all processed following the procedures described in the references. Approx. 10 s of continuous visible-light soaking at 10 mW cm^–2^ (0.1 J cm^–2^) was typically required for these PL spectral changes. We postulate that previously reported PL studies on mixed-halide perovskites,^15,17,18^ in many cases done with ultrafast pulsed excitation, may have used much smaller light-soaking dosages that were insufficient to produce these changes.

## Conclusions

We have observed the formation of a new low energy PL feature upon light soaking of (MA)Pb(Br_*x*_I_1–*x*_)_3_ and other mixed-halide perovskites. This spectral change, accompanied by the growth of sub-bandgap absorption states and a splitting of XRD peaks, is consistent with photo-induced halide segregation. In the case of (MA)Pb(Br_*x*_I_1–*x*_)_3_ solar cells, the red-shift in PL upon light illumination indicates a reduction in the electronic bandgap and quasi-Fermi level splitting, reducing their achievable *V*_OC_'s. This photo-induced instability is expected to have implications for the operation and reliability of other optoelectronic devices made from this family of materials. Amplified stimulated emission has been recently demonstrated from a few compositions in the (MA)Pb(Br_*x*_X_1–*x*_)_3_ (X = Cl and I) families using sub-ns pulsed optical excitation.^18,19^ Photo-induced changes in PL will likely need to be suppressed in order to achieve stable continuous-wave lasing from these materials. We recently suggested that ion migration could play a role in perovskite photovoltaic hysteresis.^34^ Further studies on the mechanism of light-induced trap formation may be crucial in improving the performance and stability of hybrid mixed-halide perovskite photovoltaics and optoelectronic devices. These reversible, light-induced transitions may also enable applications in optical memory and switching.
