# Supplementary material for: Reversible photo-induced trap formation in mixed-halide hybrid perovskites for photovoltaics
Source: Chem Sci. 2014 Nov 4;6(1):613–7. doi: 10.1039/c4sc03141e (PMC5491962; doi:10.1039/c4sc03141e)
Supplement: Supplementary file 1 [file SC-006-C4SC03141E-s001.pdf]

This journal is © The Royal Society of Chemistry 2014

Electronic Supplementary Material

Reversible photo-induced trap formation in mixed-halide hybrid perovskites for photovoltaics†

Eric. T. Hoke,[a] Daniel J. Slotcavage,[a] Emma R. Dohner,[b] Andrea R. Bowring,[a]
Hemamala I. Karunadasa,[b*] and Michael D. McGehee[a**]

[a] Department of Materials Science and Engineering, Stanford University, Stanford, California 94305, United States.
[b] Department of Chemistry, Stanford University, Stanford, California 94305, United States.
* hemamala@stanford.edu ** mmcgehee@stanford.edu

Methods

Film and device fabrication: $(MA)Pb(Br_xI_{1-x})_3$ was deposited by spincasting from 0.55 M solutions in DMF made by mixing varying ratios of equimolar solutions of 1) $PbBr_2$ + $(CH_3NH_3)Br$ and 2) PbI_2 + $(CH_3NH_3)I$. Methylammonium iodide[1] and methylammonium bromide[2] were synthesized and purified as described in the references herein. We observed similar transient behaviour in the PL of perovskite films under illumination that were made using commercially available methylammonium iodide and methylammonium bromide from Dyesol. For optical and XRD characterization, the thin films were deposited on glass slides or silicon substrates and annealed at 100 ˚C for 5 minutes in dry air, followed by spincoating of a ~500 nm thick polystyrene layer from chlorobenzene to protect the perovskite from moisture exposure. We observed the same PL spectra and changes in the PL spectra under illumination from the perovskite films without the polystyrene layer that were tested in a nitrogen environment. Devices were fabricated with the structure FTO/ compact TiO_2/ mesoscopic TiO_2 / perovskite / doped-spiro-OMeTAD / Gold. The perovskite layer was deposited by spincoating 0.55 M $(CH_3NH_3)Pb(Br_xI_{1-x})_3$ solutions in DMF at 2000 rpm for 60 seconds followed by annealing at 100 ˚C for 5 minutes in dry air. The other material layers were deposited following published procedures.[3]

$(MA)Pb(Br_xI_{1-x})_3$ single crystals: Crystals of $(MA)PbBr_3$ (orange, rhombic dodecahedra) were first grown by combining stoichiometric ratios of $PbBr_2$ and (MA)Br in concentrated (9 M) hydrobromic acid. Diffusion of acetone into this solution afforded crystals within 2 days, which were filtered and dried under reduced pressure. These $(MA)PbBr_3$ crystals (0.04 g) were then

added to 0.8 mL of acetonitrile. To tune the halide composition, differing ratios of concentrated hydroiodic acid (5 M) and hydrobromic acid (9 M) (totaling 200 μL) were then added to the acetonitrile, resulting in the complete dissolution of the $(MA)PbBr_3$ crystals. Diffusion of diethyl ether into this solution yielded crystals (square plates, ranging from orange to black depending on iodide content) after 1 day, which were filtered and dried under reduced pressure. The final halide composition was estimated by comparing the reflectance spectra of the single crystals to that of the thin films for various composition.

Absorption: The absorption coefficient was calculated using the formula $\alpha = \frac{\ln\left(\frac{1-R}{T}\right)}{t}$ where R is the specular reflection and T is the specular transmission (measured using a Jasco integrating sphere and UV-Vis spectrometer) and t is the thickness (measured with a Dektak profilometer) of the thin film on glass. The measurement was performed on films with thicknesses of ~400 nm and ~70 nm to minimize impact of light scattering and surface roughness over a larger measurement range.

Photoluminescence (PL) was measured using a spectrograph (Acton Research SpectraPro 500i) equipped with a Hamamatsu silicon CCD array detector and was corrected for the instrument response and the Jacobian for converting from wavelength to energy. The materials were excited with the 457 nm line of an argon ion laser unless stated otherwise.

Photocurrent spectroscopy (i.e. external quantum efficiency) measurements were performed on photovoltaic devices using monochromatic light (<10 μW/cm^2) optically chopped at 3 Hz. The generated photocurrent current was measured using a lock-in detector (Stanford Research Systems 830). To convert the photocurrent spectroscopy data taken below the band edge to an absolute absorption coefficient, the photocurrent was scaled by a constant factor to match the absorption coefficient determined from integrating sphere measurements.

X-ray Diffraction θ–2θ measurements were performed on perovskite thin films on silicon wafers or glass using a Panalytical X'Pert Pro Diffractometer (Copper anode, Kα1 =1.54060 Å, Kα2 = 1.54443 Å, Kα2 / Kα1 ratio = 0.50).

X-ray Diffraction Peak width analysis:

The XRD patterns were first corrected to remove the contribution from copper Kα2. The peak diffracted angle (θ) full width at half maximum (FWHM) breadth B was then corrected for the instrumental response (IR) according to Equation 1.

$$B = \sqrt{(\Delta\theta_{FWHM})^2 - (\Delta\theta_{IR,FWHM})^2} \tag{1}$$

Assuming that the peak broadening is due only to crystallite-size and strain effects, the y-intercept of a linear fit on a Williamson-Hall plot is inversely proportional to the crystallite grain diameter (D) and the slope is equal to the average uncorrelated strain (ε) according to equation 2.[4,5]

$$\frac{B\cos(\theta)}{\lambda} = \frac{K}{D} + \frac{4\sin(\theta)}{\lambda}\varepsilon \tag{2}$$

Here λ represents the x-ray wavelength. We assume a proportionality constant of $K = 0.94$, which is appropriate if the crystallites are roughly spherical in shape.

Photothermal deflection spectra (PDS) relies on the complete or fractional conversion of absorbed electromagnetic radiation by the material of interest into heat via non-radiative de-excitation processes. This conversion process causes a temperature rise in the material itself and its surroundings. The temperature rise leads to a localized change of the index of refraction in the surrounding deflection medium, which is measured and correlated to the absorption coefficient of the material of interest. Sample films were spin-cast from DMF onto quartz substrates, annealed at 100 ˚C for 5 minutes and immersed in a cuvette filled with Fluorinert TM (3M). The samples were pumped with the monochromated and collimated output of a halogen lamp. A second (transverse) He:Ne laser probe laser beam is deflected by the localized change of the refractive index of the surrounding deflection medium, and a position sensitive detector records the periodic deflection by a lock-in technique. The measured deflection is proportional to the absorption coefficient of the measured thin film at low deflection angles.

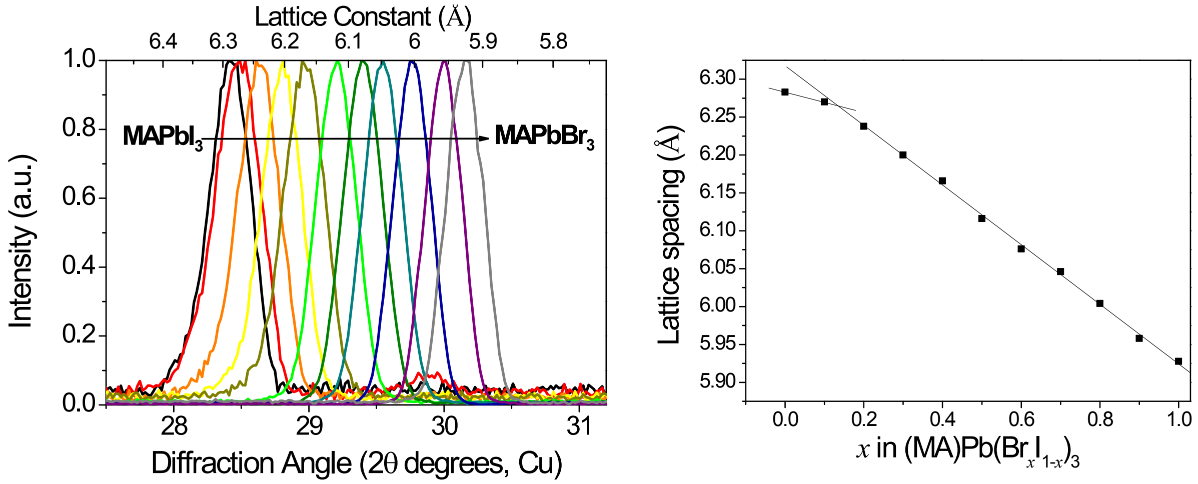

Figure S1. (left) θ–2θ XRD patterns of $(MA)Pb(Br_xI_{1-x})_3$ thin films showing the 220 (for $x \leq 0.1$) and 200 (for $x \geq 0.2$) diffraction peaks and (right) the pseudo-cubic lattice parameter extracted from the XRD pattern as a function of alloy ratio.

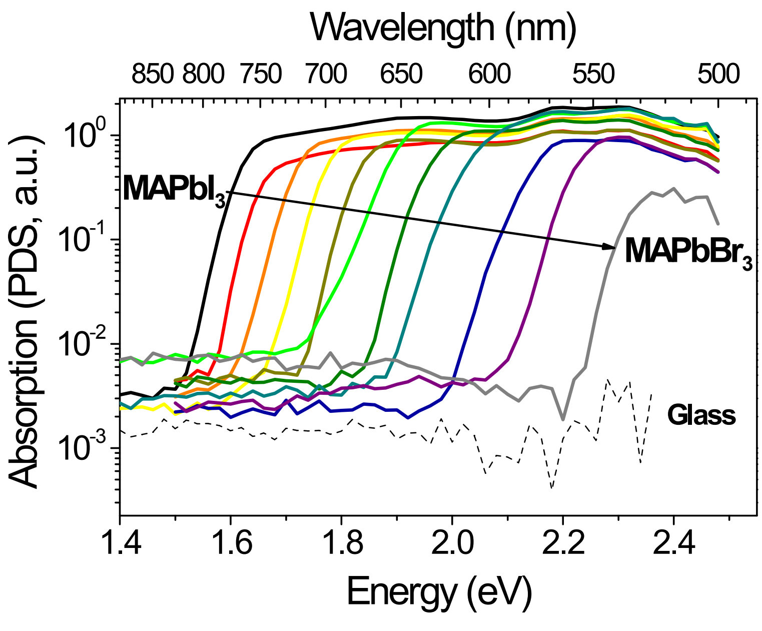

Figure S2. Photothermal deflection spectra (PDS) of $(MA)Pb(Br_xI_{1-x})_3$ thin films, demonstrating a more gradual absorption onset for the $x = 0.5$ (light green) composition compared to the other alloys.

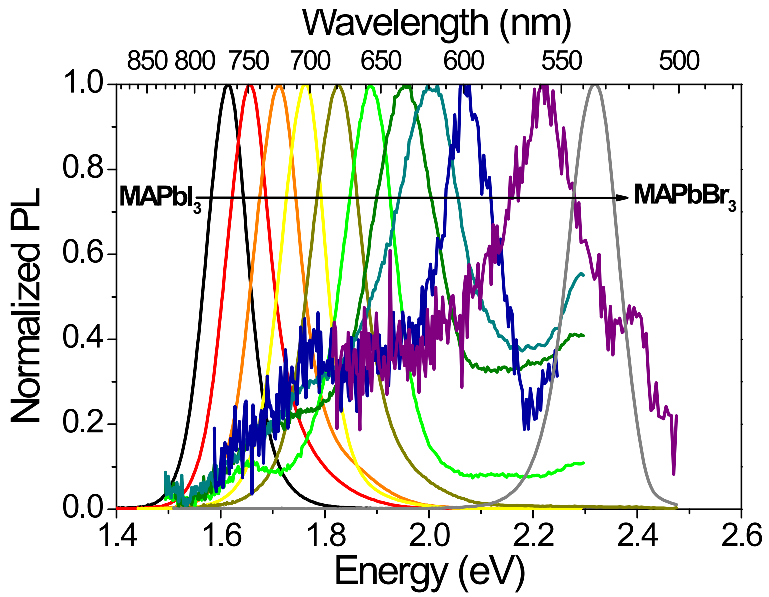

Figure S3. Initial photoluminescence spectra of $(MA)Pb(Br_xI_{1-x})_3$ thin films before noticeable photo-induced spectral changes have occurred. We note that the noise level is high for the mixed-halide films with highest bromine content, as low excitation powers and short integration times were required to capture the initial PL spectra before photo-induced spectral changes occurred in these films.

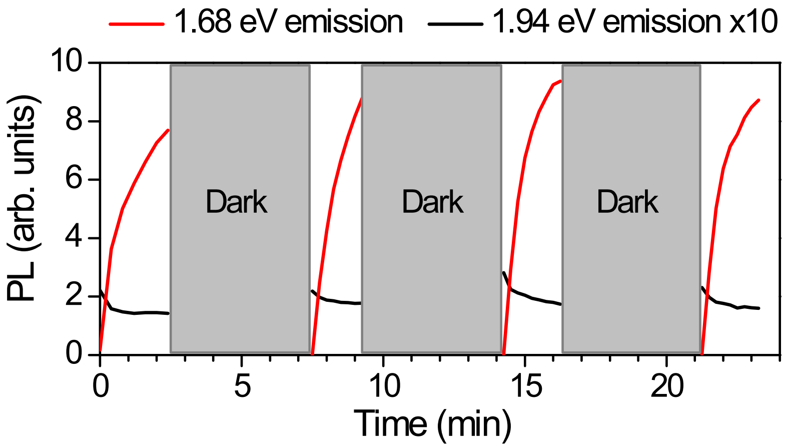

Figure S4. PL emission intensity vs. time of a $(MA)Pb(Br_{0.6}I_{0.4})_3$ (x=0.6) thin film at the two emission peaks. The film was excited at 457 nm, 15 mW/cm^2.

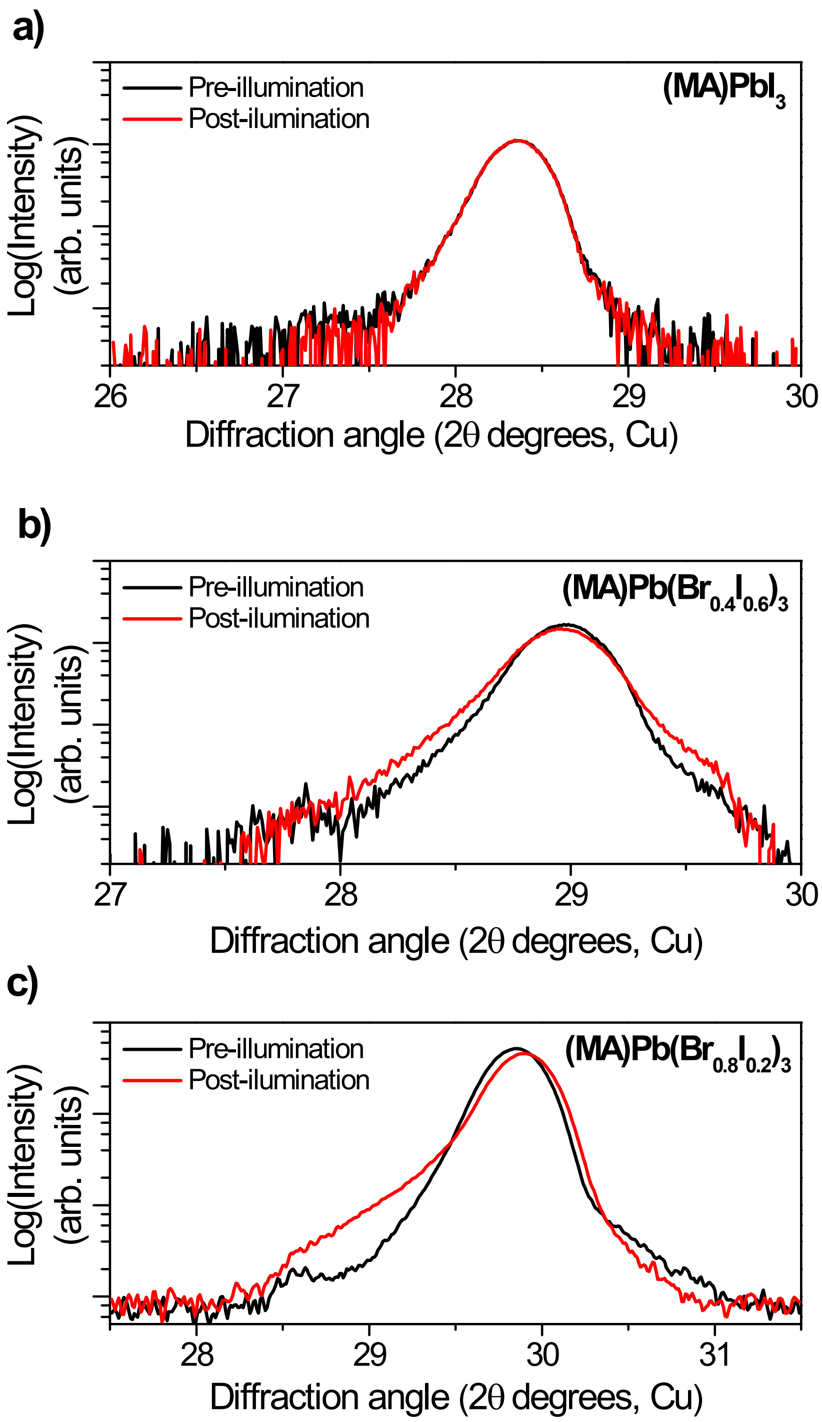
a)
Pre-illumination
Post-ilumination
(MA)PbI3
Log(Intensity)
(arb. units)
26
27
28
29
30
Diffraction angle (2θ degrees, Cu)
b)
Pre-illumination
Post-ilumination
(MA)Pb(Br0.4I0.6)3
Log(Intensity)
(arb. units)
27
28
29
30
Diffraction angle (2θ degrees, Cu)
c)
Pre-illumination
Post-ilumination
(MA)Pb(Br0.8I0.2)3
Log(Intensity)
(arb. units)
28
29
30
31
Diffraction angle (2θ degrees, Cu)

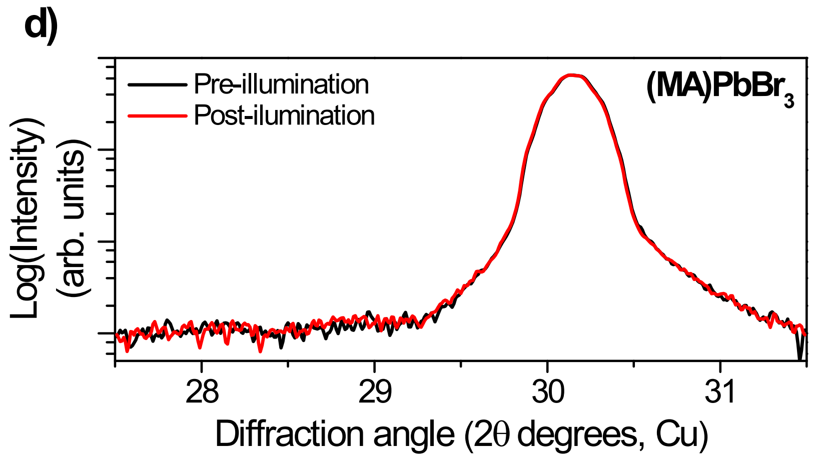

Figure S5. XRD pattern of (a) the 220 peak of (MA)PbI_3 and the 200 peak of (b) $x = 0.4$, (c) $x = 0.8$ and (d) $x = 1$ (MA)Pb(Br_xI_{1-x})$_3$ thin films, before (black) and after (red) white light (LED) soaking for 5 minutes at ~50 mW/cm^2.

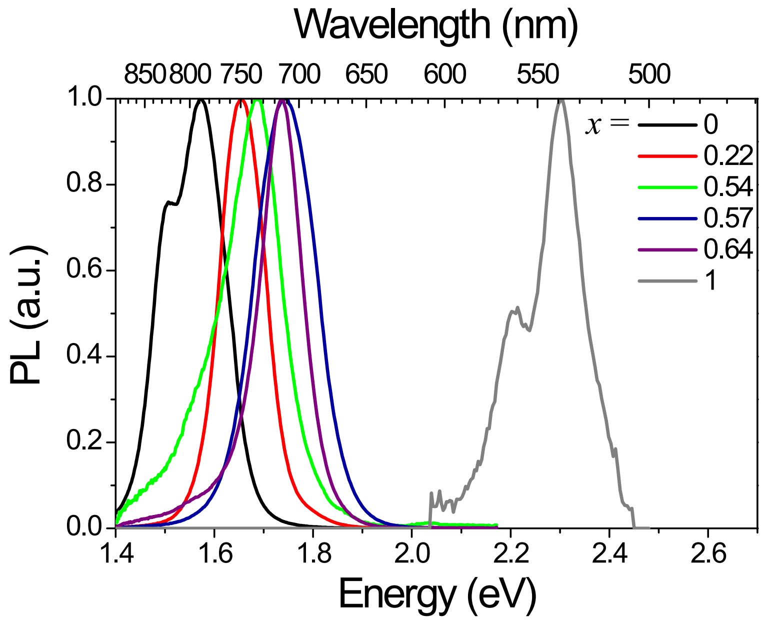

Figure S6. Photoluminescence spectra of single crystals of (MA)Pb(Br_xI_{1-x})$_3$. The halide ratio, x, was determined from inductively-coupled plasma mass spectrometry (ICP-MS) measurements on the single crystals in 0.5% nitric acid solutions using a Thermo XSeries II ICP-Mass Spectrometer. The excitation beam was tightly focused in order to obtain sufficient signal from the small crystals and consequently we were unable to observe the PL spectra before some light soaking effects had already taken place.

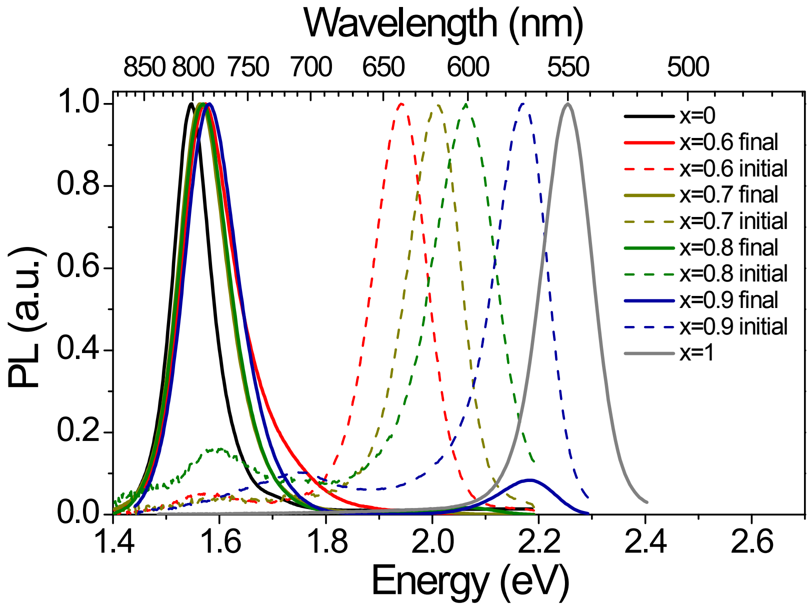

Figure S7. Photoluminescence spectra of thin films of mixed-halide formamidinium lead perovskites $(HC(NH_2)_2)Pb(Br_xI_{1-x})_3$ spun from DMF, before and after light soaking (457 nm excitation). The halide composition was tuned by mixing varying ratios of equimolar solutions of 1) $PbBr_2$ + $(HC(NH_2)_2)Br$ (purchased from Dyesol) and 2) PbI_2 + $(HC(NH_2)_2)I$ (purchased from Dyesol). The formation of a low-energy PL peak with light soaking was also observed in the iodide-rich films ($x < 0.6$). However, we were not able to obtain a single crystalline phase (in the dark, pre-illumination) with these materials (as measured by XRD).

References:

1 M. M. Lee, J. Teuscher, T. Miyasaka, T. N. Murakami, and H. J. Snaith, *Science*, 2012, **338**, 643–7.

2 J. H. Noh, S. H. Im, J. H. Heo, T. N. Mandal, and S. Il Seok, *Nano Lett.*, 2013, **13**, 1764–9.

3 J. Burschka, N. Pellet, S.-J. Moon, R. Humphry-Baker, P. Gao, M. K. Nazeeruddin, and M. Grätzel, *Nature*, 2013, **499**, 316–9.

4 G. K. Williamson and W. H. Hall, *Acta Metall.*, 1953, **1**, 22–31.

5 V. Mote, Y. Purushotham, and B. Dole, *J. Theor. Appl. Phys.*, 2012, **6**, 6.
